# Fabrication, Characterization and Cytotoxicity of Spherical-Shaped Conjugated Gold-Cockle Shell Derived Calcium Carbonate Nanoparticles for Biomedical Applications

**DOI:** 10.1186/s11671-017-2411-3

**Published:** 2018-01-02

**Authors:** Hanan Karimah Kiranda, Rozi Mahmud, Danmaigoro Abubakar, Zuki Abubakar Zakaria

**Affiliations:** 1 Laboratory of Molecular Biomedicine, Institute of Bioscience, Universiti Putra Malaysia, 43400 UPM, Serdang, Malaysia; 2 Department of Imaging, Faculty of Medicine and Health Sciences, Universiti Putra Malaysia, 43400 UPM, Serdang, Malaysia; 3 Department of Veterinary Preclinical Sciences, Faculty of Veterinary Medicine, Universiti Putra Malaysia, 43400 UPM, Serdang, Malaysia

**Keywords:** Au-CSCaCO_3_NPs, Biomedical applications, Calcium carbonate nanoparticles, Characterization, Cytotoxicity, Fabrication and gold nanoparticles

## Abstract

The evolution of nanomaterial in science has brought about a growing increase in nanotechnology, biomedicine, and engineering fields. This study was aimed at fabrication and characterization of conjugated gold-cockle shell-derived calcium carbonate nanoparticles (Au-CSCaCO_3_NPs) for biomedical application. The synthetic technique employed used gold nanoparticle citrate reduction method and a simple precipitation method coupled with mechanical use of a Programmable roller-ball mill. The synthesized conjugated nanomaterial was characterized for its physicochemical properties using transmission electron microscope (TEM), field emission scanning electron microscope (FESEM) equipped with energy dispersive X-ray (EDX) and Fourier transform infrared spectroscopy (FTIR). However, the intricacy of cellular mechanisms can prove challenging for nanomaterial like Au-CSCaCO_3_NPs and thus, the need for cytotoxicity assessment. The obtained spherical-shaped nanoparticles (light-green purplish) have an average diameter size of 35 ± 16 nm, high carbon and oxygen composition. The conjugated nanomaterial, also possesses a unique spectra for aragonite polymorph and carboxylic bond significantly supporting interactions between conjugated nanoparticles. The negative surface charge and spectra absorbance highlighted their stability. The resultant spherical shaped conjugated Au-CSCaCO_3_NPs could be a great nanomaterial for biomedical applications.

## Background

The production of monodisperse nanoparticles has emerged significant in electronic, optical, biomedical, and magnetic applications [1–4]. Their evolution and that of biomaterials has favorably enhanced pharmaceuticals [5], biomedical systems [6], drug delivery systems [7], cosmetics, and water treatment [7–9]. In the same regard, the development of conjugated materials that are biocompatible, biogenic, and nontoxic could have valuable contributions to the fields of bioscience and biomedicine [10]. Additionally, biocompatible metallic conjugated bio and nanomaterial could contribute to more scientific advancements for biomedical applications such as tissue engineering [5], therapeutics [11], and drug delivery [12]. This has been shown in recent works elaborately, like the use of injectable self-assembling collagen-gold hybrid hydrogel [13], colloidal gold-collagen core-shell nanoconjugates [14], and co-assembled carrier-free nano drugs for antitumor therapy [15]. A number of studies have also documented that metallic nanoparticles can produce enzyme electrodes in electrochemical biosensors with inorganic non-silica porous materials [16]. Furthermore, the synthesized graphene oxide-albumin nano-hybrids have also displayed their potential benefit towards enhanced photodynamic therapy [17]. Altogether, this has only sparked more interest with other possible applications such as biomedical imaging and bio-sensory systems [16, 18].

Calcium carbonate as a raw, natural mineral has been used in a wide range of applications including biomedical, industrial, and nanotechnology [10, 19–21]. Aragonite as a calcium carbonate polymorph richly exists in cockle shell (*Anadara granosa*), a molluscs popularly, also found in Malaysia [22]. Aragonite is biogenic unlike the other calcium carbonate polymorphs of calcite and vaterite, making up to 95–98% of cockle shell. Calcium carbonate, an inorganic material of aragonite polymorph, naturally and commonly exists within the cockle shells [23]. Aragonite polymorph has increasingly attracted attention in research field due to its biocompatibility properties and promising potential in the development of anticancer drug delivery systems [24] and biomedical imaging [25, 26]. Currently, most of prior research studies have revealed mainly two methods of production of calcium carbonate [26]. They include the co-precipitation or double decomposition and carbonation of CO_2_ gas through calcium hydroxide under controlled settings, which regrettably none produces biogenic calcium carbonate [26–28]. Therefore, the products contain a mixture of calcite and vaterite in high quantities which are unsuitable for biomedical use because of their non-biocompatibility and high toxicity reports [26].

However, with the increasing use of nanotechnology in biomedical applications, the present study is focused on the synthesis of controlled cockle shell-derived calcium carbonate nanoparticles (CSCaCO_3_NPs) with unique size and shape using dodecyl dimethyl betaine (BS-12) [29]. This is inspired by prior works that utilize BS-12 as bio mineralization catalyst in the synthesis of CSCaCO_3_NPs that can easily be manipulated for bio-applications, cost efficient, and relatively pure nanoparticles [30]. The morphology and size of synthesized nanoparticles are crucial in determining their physicochemical properties, with focus on metal nanoparticles given their vast potential biomedical applications [31]. Gold nanoparticles (AuNPs) have continuously been used due to their optical properties, different size range, and color which are dependent on absorption maxima variations or the synthesis method employed [32]. AuNPs’ size and shape affect their absorption and emission characteristics in the light visible spectrum, making them vary from visible to near infrared regions. Therefore, due to their synthesis [33], physicochemical properties [34], biocompatibility [35], and surface functionalization [36], they can be manipulated for different and particular applications [37]. In addition, it also has been stated that in medical diagnostics, they are not completely used and their value possibly obscure [37].

So perhaps upon appropriate functionalization, they could be redesigned for cancer imaging [38], cancer treatment [39], drug delivery [40], and sensory gadgets [41]. A coating is essential to fabricate nano-hybrid biomaterial with functionalized properties like gold nanoparticles (AuNPs) conjugated with porous calcium carbonate nano-spheres [16, 42]. The resultant conjugated gold-calcium carbonate nanomaterial or nano-composite hybrid, which could retain the advantageous parental traits such as biocompatibility, good solubility, and dispersibility in solution [16]. Conjugated gold nanoparticles that exhibit strong color change and localized surface plasmon resonance (LSPR) could be excellent candidates for potential multiple receptor systems such as aptamers, peptides, and antibodies [35, 43–45]. The fabrication of water-soluble conjugated polymers and its applications in biosensors, fluorescence imaging, and drug delivery have been successfully realized [46–48]. However, the conjugated nanoparticles or nanomaterial has progressively improved advantages such as photo stability [48, 49] and low cytotoxicity [50] over the years except for friendlier preparation [51] and separation features [48].

Herewith, the AuNPs and CSCaCO_3_NPs are controllably synthesized and used to fabricate and characterize biogenic conjugated gold-cockle shell-derived calcium carbonate nanoparticles (Au-CSCaCO_3_NPs) whose diameter size ranges from 19–51 nm. Initially, the AuNPs preparation is inspired by the classic Turkevich method [52] and the cockle shell derived nanoparticles using the dodecyl dimethyl betaine synthetic approach [26]. The modifications in the synthetic parameters such as concentration could proficiently decrease or increase their size. Consequently, the synthesized nanomaterial was characterized and investigated for cytotoxicity. The Au-CSCaCO_3_NPs preparation added advantages are; easy synthesis and cost efficiency.

## Methods/Experimental

### Materials and Chemical Reagent

The gold salt (tetra chloroauric acid containing 49% gold solution) and the tri-sodium citrate were purchased from prima nexus Sdn Bhd (Malaysia). Fresh cockle shell was obtained from local market (Pasar borong, Seri Kembangan, Selangor, Malaysia). Dodecyl dimethyl betaine (BS-12) and indocyanine green dye (ICG) were purchased from Sigma-Aldrich (Steinheim, Germany). Dulbecco’s modified Eagle’s medium (DMEM), fetal bovine serum (FBS), antibiotics combination (glutamine 100 mmol/L, penicillin 100 U/mL, and streptomycin 100 μg/mL), phosphate-buffered saline (PBS), dimethyl sulfoxide (DMSO), and MTT (3-Dimethylthiazo-2, 5-diphynyltetrazolium Bromide dye) were purchased from Naclai tesque, Inc., Kyoto, Japan. All other reagents used were of analytical grade.

### Synthesis of Gold Nanoparticles

The synthesis was achieved using a method earlier described by Verma et al. [53] with slight modifications in concentrations, 1% tetra chloroauric acid containing 49% gold solution. Approximately, 0.1% of the gold solution was prepared and diluted in a series of concentrations of 15, 25, and 20 mM in different conical flasks, respectively. The solutions were then heated at 100 °C on a hot plate coupled with the magnetic stirring (6 positioned, WiseStir ® Korea). Then, about 1% tri-sodium citrate was added to the boiling solution with continuous magnetic stirring until color transition (yellowish gold solution turned colorless then to black then finally turned brilliant red) was observed. The heat was turned off after 15 min and allowed to cool at room temperature. The synthesized gold nanoparticles were then stored at − 4 °C for further use. The reaction was shown in the equation below:

$$ 2{\mathrm{H}\mathrm{AuCl}}_4+3{\mathrm{C}}_6{\mathrm{H}}_8{\mathrm{O}}_7\left(\mathrm{citric}\  \mathrm{acid}\right)\to 2\mathrm{Au}+3{\mathrm{C}}_3{\mathrm{H}}_6{\mathrm{O}}_5\left(3-\mathrm{ketoglutaric}\  \mathrm{acid}\right)+8\mathrm{HCl}+3{\mathrm{C}\mathrm{O}}_2 $$

### Preparation and Synthesis of Cockle Shell-Derived Calcium Carbonate Nanoparticles (CSCaCO_3_NPs)

Three kilograms of freshly obtained cockle shells were thoroughly cleaned, scrubbed, and washed. The cockle shell powder was produced according to the method described by Islam et al. [54]. The cleaned cockle shell was dried in an oven (Memmert UM500, GmbH Co, Germany) at 50 °C for 7 days. The cockle shells were ground into powder using a blender (Blender HCB, 550, USA) and sieved with a stainless laboratory test sieve (Endecott Ltd., made in London, England) with the aperture of 90 μm to obtain micron-size powder. The powder was dried for 7 days at 74 °C in the oven. The powder was further packed in airtight polythene plastic bag for later use. The cockle shell-derived calcium carbonate nanoparticles were synthesized according to the approach described by Islam et al. [55], with slight modifications to the method and synthesis parameters. Two grams of cockle shell powder were taken into 250 ml conical flask followed by 50 ml of double deionized water, and a concentration of 0.5 ml of BS-12 was added into the conical flask. The mixture in the conical flask was vigorously stirred at 1000 rpm, with a temperature of 50 °C for 135 min using a systematic multi-hotplate and magnetic stirrer with small magnetic bar. The prepared sample was separated from the mother liquid using double ring filter paper of size 125 mm (Filtres Fioroni, China). The residue was then washed thoroughly to remove the excess BS-12. The final products, CSCaCO_3_NP powder, were packed in dry-clean container and dried for 3 days (Oven Memmert UM500, GmbH Co, Germany) at 74 °C. The container was properly wrapped and sealed with Para film after addition of multiple small marble balls inside. The container was placed on a Programmable roller-ball mill (BML-6, Wisemix ® Korea) at speed of 200 rpm for 5 days. The sample was stored in airtight polythene in oven for further use.

### Synthesis of Conjugated Gold-Cockle Shell-Derived Calcium Carbonate Nanoparticles (Au-CSCaCO_3_NPs) and Inco-operation of Near Infrared (NIR) Dye

In this procedure, 0.2 g of CSCaCO_3_NPs and 5 mg of near infrared (NIR) Indocyanine green dye (ICG) were dispersed in 20 ml of gold colloid solution (pH 7) (AuNPs-solution), as similarly described by Cai et al. [16], in a clean empty conical flask. Further synthesis modifications were made, where the sample was sonicated for 20 min and incubated on magnetic stirrer with a small magnetic bar at 200 rpm for 3 days. The sample was ultra-centrifuged at a speed of 10,000 rpm for 10 min to obtain light-green-purplish, Au-CSCaCO_3_NP composite. The supernatant was decanted and pellet washed with a series of deionized water. The prepared composite material was dried in the oven for 4 days and stored in airtight polythene in oven for further analysis.

### Characterization of Conjugated Gold-Cockle Shell-Derived Calcium Carbonate Nanoparticles (Au-CSCaCO_3_NPs)

The particle size and morphology of the nanomaterial was analyzed using transmission electron microscope (TEM). The nanomaterial was dispersed in absolute alcohol and sonicated for 40 min. Approximately, 5 μl of the suspended sample solution was pipetted out on to copper grip specimen mount. The sample was viewed under TEM (Hitachi H-7100). The field emission scanning electron microscope (FESEM) (Model JOEL 7600F) operated at voltage of 5 KV and equipped with energy dispersive X-ray spectroscopy unit (EDX). This was used to characterize the surface features of the Au-CSCaCO_3_NPs. The material was dispersed in absolute alcohol and sonicated for 1 h. About 50 μl of the suspended sample solution was pipetted out on to copper grip specimen mount, dried overnight, and scanned using the electron beams. In addition, the Fourier transform infrared spectrometer (FTIR) was also used for functional analysis of the synthesized conjugated nanomaterial; the nano material was calibrated in 1 wt% in Ker (FTIR Model 100, Perkin Elmer) in the range of 400–4000 cm^−1^. Furthermore, analysis for the synthesized nano conjugate size and zeta potential was done using zetasizer (Nano ZS, Malvern Instruments). The material was suspended in deionized water and sonicated for 50 min; the homogenous suspension was deposited in the zetasizer cuvette and examined for particle size and zeta potential. The presence of different analytes of the conjugated nano composite was monitored using Uv-Vis spectrophotometer (UV - 2600) at different wavelength ranging from 300 to 800 nm.

### Cell Culture and Cytotoxicity Studies

Human breast adenocarcinoma cell line (JCRB: MCF-7) and the mouse fibroblast cell line (JCRB: NIH3T3) were cultured in DMEM (high glucose) supplemented with 10% FBS and antibiotics combination (glutamine 100 mmol/L, penicillin 100 U/mL, and streptomycin 100 μg/mL). The culture flasks (Eppendorf culture T-25 and T-75) were incubated in 5% carbondioxide at 37 °C, and cells at 80–90% confluence were used for seeding and treatment process.

#### Cells Seeding and Treatment

The cells were seeded into 96-well sterile plates at a density of 5 × 10^3^ cells per well and incubated for 24 h overnight. The media in each well were removed, and the cells were treated and co-cultured in replicates with conjugated nano composite suspension (Au-CSCaCO_3_NP) for a period of 24, 48, and 72 h. After treatment exposure was completed, the media in the wells were aspirated and washed with PBS before they were replaced with another fresh media prior to experimental treatments.

#### Preparation of Au-CSCaCO_3_NPs for Treatment

Stock solution of Au-CSCaCO_3_NPs at a concentration of 1 mg/ml in 10% serum free DMEM media was prepared. After cell seeding of MCF-7 cells and NIH3T3 cells in 96-well plates, the plates were treated and incubated with different concentrations in microgram (100–1.56) of the Au-CSCaCO_3_NPs solutions.

#### (MTT) 3-Dimethylthiazo-2, 5-diphynyltetrazolium Bromide Reagent Preparation and Protocol

Typically, 5 mg of MTT reagent powder was dissolved in 1 ml of PBS facilitated by sonicator vortex for uniform mixture. After cell seeding and treatment, the well plates were cleared and 20 μl of MTT reagent was added to each well. Immediately after, the plates were allowed to incubate for 3–4 h to allow binding of the MTT to the mitochondria of the cells. After incubation, 1 ml of DMSO was added to each of the wells which released the color product into the solution. The plates were kept in a dark room for 30 min, and optical density (OD) of the solution was measured with a micro plate reader at wavelength of 570 nm [56]. The experiments were conducted in triplicates for each cell line, and the mean values were recorded. The percentage of cell viability was determined using the formula below.

$$ \mathrm{Percentage}\  \mathrm{of}\  \mathrm{cell}\  \mathrm{viability}=\left(\ A\  Sample/A\  Control\right)\times 100 $$

where *A*_Sample_ was average OD reading of different incubated treated cells of both cell lines and *A*_Control_ was average OD reading of the different incubated cells in complete culture media only. The cytotoxicity of the cells was then assessed from the average triplicate values and exhibited as mean ± standard deviation (SD).

### Statistical Analysis

Statistical data analysis were done using SPSS software (Version 10, Chicago, USA). The experiments were done in triplicates and expressed as mean ± standard deviation (M ± SD). The significance threshold was *p* < 0.01.

## Results and Discussion

### Physicochemical Properties of the Conjugated Au-CSCaCO_3_NPs

#### Transmission Electron Microscope

**Fig. 1:**
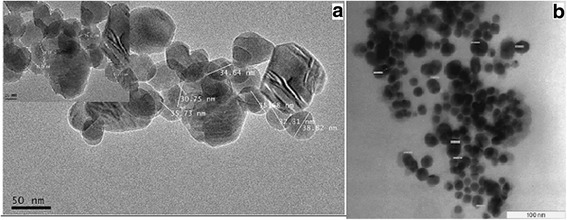
TEM (**a**, **b**) images of the Au-CSCaCO_3_NPs characterizing their different size of the nanoparticles

The purpose of the TEM micrographs was to assess the size of the synthesized conjugated Au-CSCaCO_3_NPs which show well dispersed nanoparticles with average diameter size of 35 ± 16 nm within the range of (19–51 nm). The differences in size attributed to the synthesis conditions were as shown in Fig. 1.

TEM micrographs of the nano conjugate showed ranging diameter of 19–51 nm and dispersed nanoparticles. The uniquely obtained nano-size could be attributed to the controlled synthetic conditions employed. Another possible explanation for the nanoparticle dispersity could be due to the negatively charged layer of citrate ions which aided in the repulsions of nanoparticles from each other and also, due to electrostatic repulsion and the conjugate hydration surface layer preventing aggregation and increasing conjugate stability as similarly reported by Jazayeri et al. [56]. Furthermore, the citrate capping reagent plays a role in the synthesis, which allowed for more dispersity and stability of the nanoparticle conjugate as reported by Rawat et al. [57]. The unique particle size showed the different absorbed gold nanoparticles inside calcium carbonate nano-sphere matrix similar to work done by Cai et al. [16], contributing to the observed resulting particle size shown. However, this result also confirms reports that calcite has poor ability to accommodate gold nanoparticles [16].

#### Field Emission Scanning Electron Microscopy (FESEM) and Energy Dispersive X-ray Spectroscopy (EDX)

**Fig. 2:**
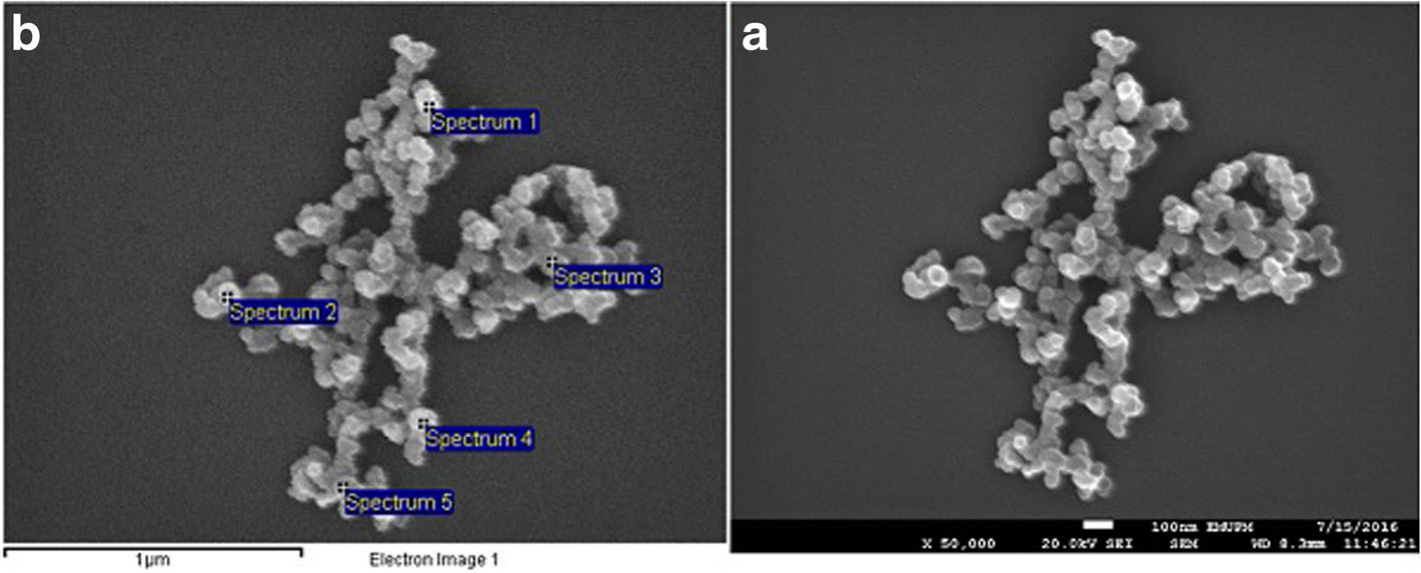
FESEM **a** FESEM micrograph of the Au-CSCaCO_3_NPs describing the morphology. **b** EDX spectra of the Au-CSCaCO_3_NPs

The FESEM micrograph assessed the morphology and shape of the synthesized nanoparticles which shows spherically shaped and chain-like Au-CSCaCO_3_NPs nanoparticles with a small degree of aggregation as displayed in Fig. 2. The elementary spectra (Fig. 2b) analyzed the elemental composition of the conjugated nanoparticles which displays 64.98% carbon, 13.53% oxygen, 0.02% calcium, 17.63% copper, and 3.85% gold as presented in Table 1.

**Table 1:** EDX elemental composition profile of the Au-CSCaCO_3_NPs

Spectrum	C	O	Ca	Cu	Au	Total
Spectrum 1	58.39	12.04	0.00	21.30	8.27	100.00
Spectrum 2	62.45	13.44	0.08	18.59	5.44	100.00
Spectrum 3	64.30	13.19	0.00	17.51	5.00	100.00
Spectrum 4	69.90	14.32	0.00	15.27	0.52	100.00
Spectrum 5	69.84	14.65	0.00	15.51	0.00	100.00
Mean ± SD	64.97 ± 4.95	13.53 ± 1.03	0.02 ± 0.04	17.64 ± 2.47	3.84 ± 3.51	100.00

FESEM micrographs described the unique morphology as spherical shape, smoothed surface, and chain-like structured conjugated nanoparticles whose physical or chemical properties could be explained as a result of the preparation conditions and synthetic methods [58]. Similarly the spherical structural nature displayed by the conjugate nanoparticles was similar to those reported by Verma et al. [53], but contrary to the small degree of aggregation presented. A possible account for this outcome could be due to the hydrophobic and electrostatic interactions between the gold nanoparticles and cockle shell-derived calcium carbonate nanoparticles leading to strong binding [48]. Additionally, the role of BS-12 employed in the synthesis was reflected in the breakdown of the nanoparticles to spherical shape analogous with the work documented by Islam et al. [55]. The elementary profile (Table 1) revealed no significant changes contrary to the expected result. Similarly, observed findings with the chemical composition of the conjugated nanoparticles are documented as earlier shown in prior works [26, 54].

#### Surface Charge and Size Distribution by Intensity

**Fig. 3:**
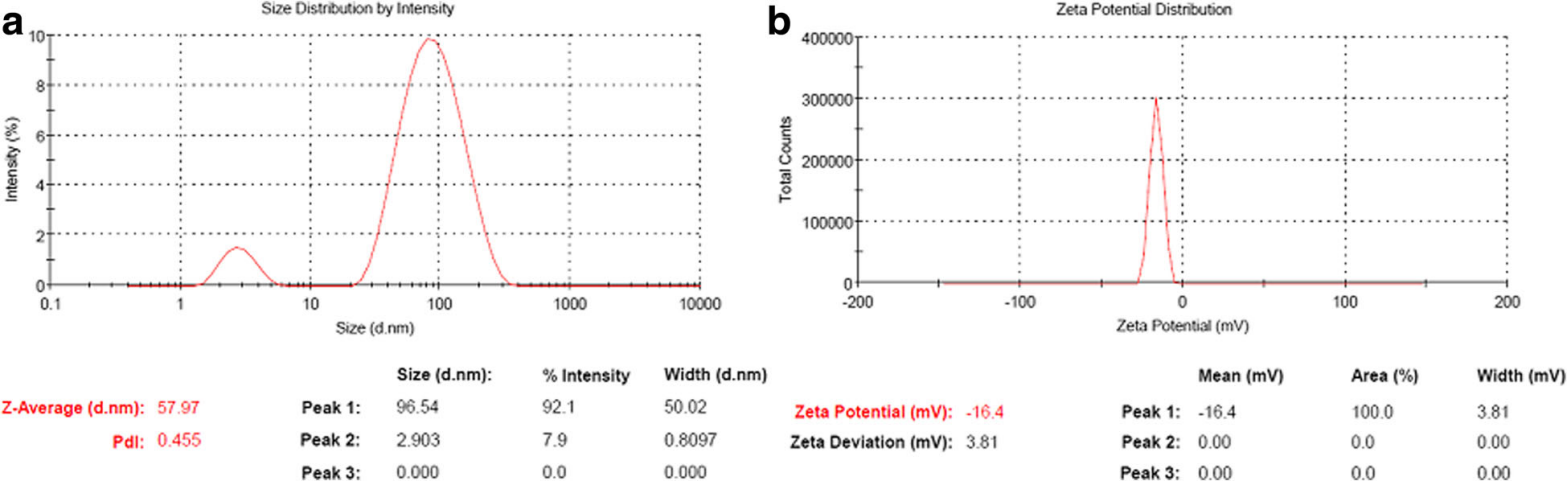
**a** Particle size distribution by intensity of the Au-CSCaCO_3_NPs. **b** Zeta potential of the Au-CSCaCO_3_NPs showing the surface charge

The zeta potential of the conjugated nanoparticles was done, in order to assess their surface charge, stability, and size distribution by intensity which reveals negative charge of − 16.4 ± 3.81 mV and conjugated nanoparticle average size of 57.97 nm as revealed in Fig. 3 and Table 2.

**Table 2:** Zeta potential of the Au-CSCaCO_3_NPs, their size distribution by intensity (d nm), and poly dispersity index (PdI) expressed in mean ± standard deviation

Au-CSCaCO_3_NPs	
Peak 1	96.54
Peak 2	2.903
Peak 3	0.000
Z-Average (d nm)	57.97
PdI	0.4
Zeta Potential (mV) ± SD	− 16.40 ± 3.81

Zeta potential is an important assay in assessing the nanoparticle surface electrostatic charge which was determined using zeta sizer. This further explained the dispersity of the nanomaterial in solution, enabling us to understand the overall stability, nanoparticle shelf life, particle interactions between the charged particles, and their implications [59]. The zeta potential assessment of the conjugated nanomaterial indicated stability of the nanoparticles at − 16.4 mV and a poly dispersity index (PdI) of less than 0.5. A possible explanation could be attributed to the presence of more electro-repulsion between the particles in suspension during measurement. Furthermore, the agglomeration tendencies may have also influenced the size distribution leading to bigger size due to the synthetic methods. Prior study by Hoque et al. has similarly documented [60] that highly positive or negative zeta potential decreases aggregation and increases stability. Additionally, the physicochemical differences of the nanoparticles synthesized could be accounted to the synthesis methods used. Kanaujia and co-workers’ [61] works have also emphasized that higher negative or positive values of zeta potential indicate stability and avert aggregation of particles, because of electric repulsion that electrically stabilizes the nanoparticles dispersion also reported by Isa et al. [62].

#### Fourier–Transform Infrared spectrometer (FTIR)

**Fig. 4:**
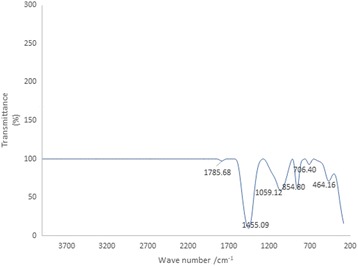
Fourier transform infrared spectrometer spectrum of the main characteristic peaks of Au-CSCaCO_3_NPs. All marks correspond to the frequencies discussed in the text

The FTIR spectrum of Au-CSCaCO_3_NPs shows that the most outstanding peak appeared at 1455.09 cm^−1^ followed by peaks observed at 1059.12 cm^−1^, 854.80 cm^−1^, and 464.16 cm^−1^, respectively. Also, weak peaks were observed at 706.40 cm^−1^ and 1785.68 cm^−1^ as presented in Fig. 4.

The FTIR spectrum of Au-CSCaCO_3_NPs as presented showed that the most outstanding peak appeared at 1455.09 cm^−1^, attesting to the oxygen-hydrogen (O–H) bonds present in carboxylic groups of gold nanoparticles [14] and cockle shell nanoparticles, followed by peaks that best showed presence of aragonite polymorph marker observed at 1059.12 cm^−1^, 854.80 cm^−1^, and 706.40 cm^−1^, which are known to report alkyl group occurring in the cockle shell-derived nanoparticles that were consistent to the spectrum peaks [55]. Similarly, the weak peak was observed at 1785.68 cm^−1^ due to the presence of carboxylic group [54], and an additional peak was observed at 464.16 cm^−1^. All the peaks showed significant characteristic of the presence of covalent bonds, carbon-carbon (C–C), carbon-oxygen (C–O), and carbon-nitrogen (C–N) linkages whose appropriate functional groups were present in our conjugated nanoparticles. The FTIR essentially identified the functional groups present, by obtaining the infrared spectrum peaks of the conjugated nanomaterial and simultaneously collecting high spectral resolution data over a wide spectral range (400–4000 cm^−1^) [63]. However, calcite polymorph of calcium carbonate is reported to have peaks ranging from 2000 to 2900 cm^−1^ with the nanoparticles fabricated by carbonation method [64].

#### Uv-Vis Spectrophotometer

**Fig. 5:**
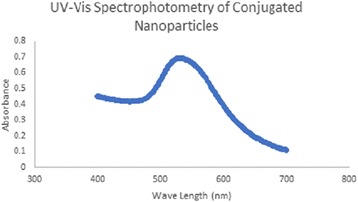
Uv-Vis spectrophotometer absorbance spectrum of the Au-CSCaCO_3_NPs as discussed in the text

The conjugated nanoparticles synthesized show a heavy absorption peak at 530 nm as shown in Fig. 5.

Gold nanostructures have a wide light absorbance due to localized surface plasmon resonance effect of AuNPs [65, 66]. A number of reports have shown that gold particles often have a sharp absorbance peak observed between 500–520 nm [66–69]. This technique allowed for further assessment of the conjugated Au-CSCaCO_3_NPs size, concentration, and aggregation level [65]. The absorbance band is also known to shift to the smaller wavelengths indicating the reduction in particle sizes, and the symmetrical shape of the absorption spectra indicates a narrow particle size distribution [70], thus confirming our conjugated Au-CSCaCO_3_NPs which displayed a wider absorption peak between 500–550 nm and highest point at 530 nm wavelength. Acceptably in the near infrared visible spectra region, at which light is easily attenuated by the tissue and absorption peak shifts significantly to longer wavelength [71]. A possible explanation for this could be due to the synthesis and conjugation of the nanomaterial. Also consistent with Srinath et al., who revealed that the position of the absorption band mostly depends on the color variation, aggregation and surface-adsorbed species [72]. Furthermore, the absorption spectrum of nanoparticles could shift depending on color, morphology, and size due to the gold plasmon resonance property [73]. Nanostructures with NIR photo thermal properties have ability to scatter light strongly, which has significant applications in biomedical imaging [74, 75].

### Cytotoxicity Studies

#### MTT (3-Dimethylthiazo-2, 5-diphynyltetrazolium Bromide)

**Fig. 6:**
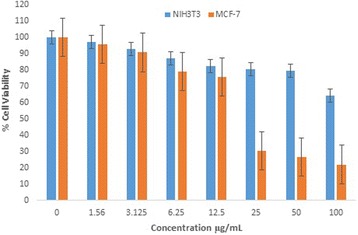
Cytotoxicity assessment of the MCF-7 and NIH3T3 treated Au-CSCaCO_3_NPs cells using MTT assay giving percentage cell viability

Cytotoxicity studies on human breast carcinoma cells (MCF-7) and mouse embryonic fibroblast cells (NIH3T3) reveal that the Au-CSCaCO_3_NPs inhibited over 70% cell proliferation causing cancer cell death and almost 40% inhibition of the fibroblast cells at 100 μg dosage. The IC_50_ and lower concentration doses such as 25 μg also proved toxic to the cancer cells revealing low cell viability and also inhibiting more than 50% cell proliferation of the cancer cells for the nanoparticles. On the other hand, identical concentration dosages to the fibroblast cells showed increased and consistent cell viability of the fibroblast cells. The IC_50_ displayed up to 80% cell viability of the fibroblast cells, as presented in Fig. 6.

3-Dimethylthiazo-2,5-diphynyltetrazolium Bromide (MTT) is a colorimetric assay acceptably used to determine cell viability [76]. Utilizing mitochondrial enzymes in the electron transport chain [77], viable cells with active metabolism converted MTT into purple-colored formazon crystals in the cellular cytosol [78]. The crystals were dissolved after cell lysis on adding an organic solvent dimethyl sulfoxide (DMSO) which is proportional to live cell number, unlike dead cells, due to cytotoxicity that are unable to carry out the reaction [79]. The conjugated nanoparticles displayed consistent cell death against the cancer cells and reliable cell viability of the fibroblast cells with concentration doses ranging from 25–100 μg. Furthermore, attesting low cytotoxicity and highlighting the biocompatibility of Au-CSCaCO_3_NPs and potential usefulness for biomedical applications, the cytotoxicity could be due to the internalization of the nanoparticles which possibly triggered intracellular responses and thus induced cellular damage because of interaction with cell organelles. Despite contrary cytotoxicity findings with works done on HeLa cells (human cervical cancer cell line) due to nanoparticles inducing oxidative damage [35, 80], Zhang et al. demonstrated the biocompatibility of the nanoparticles and its likely use for drug delivery systems [80]. Similarly, reports of gold nanoparticles confirmed nontoxic dependent on their size [81] and concentration [39]. Studies strongly confirmed that biogenic gold conjugates are stable and nontoxic nanocarrier used in biomedical application [35, 39] suggesting use for biomedical applications such as drug delivery and cancer therapy [82].

## Conclusions

Spherical-shaped conjugated gold-cockle shell-derived calcium carbonate nanoparticles (Au-CSCaCO_3_NPs) were obtained. The conjugated nanoparticles were synthesized using a simpler, environmental friendly, and cost-efficient synthetic approach. Furthermore, based on the results, the obtained conjugated nanoparticles were relatively pure and stable. The source of material used for the cockle shell-derived nanoparticles is biogenic, readily available, and naturally occurring as seawater mollusca cockle shell. Based on the presented evidences, the conjugated Au-CSCaCO_3_NPs could be a good biomaterial for biomedical applications.

## Abbreviations

Au-CSCaCO_3_NPs: Synthesized Conjugated Gold-Cockle Shell Derived Calcium Carbonate Nanoparticles
AuNPs: Gold nanoparticles
BS-12: Dodecyl dimethyl betaine
C–C: Carbon-carbon bond
C–N: Carbon-nitrogen bond
C–O: Carbon-oxygen bond
DMEM: Dulbecco’s modified Eagle’s medium
DMSO: Dimethyl sulfoxide
EDX: Energy dispersive X-ray
FBS: Fetal bovine serum
FESEM: Field emission scanning electron microscope
FRGS: Fundamental Research Grant Scheme
FTIR: Fourier transform infrared spectroscopy
HeLa cells: Human cervical cancer cell line
IC_50_: 50% inhibition concentration
ICG: Indocyanine green dye
JCRB: Japanese Collection Research Bioresource
LSPR: Localized surface plasmon resonance
MCF-7: Human breast adenocarcinoma cell line
MTT: 3-Dimethylthiazo-2, 5-diphynyltetrazolium Bromide Dye
NIH-3T3: Mouse embryonic fibroblast cell line
NIR: Near infrared
O–H: Oxygen-hydrogen bond
OD: Optical density
PBS: Phosphate-buffered saline
TEM: Transmission electron microscope
